# Supplementary material for: Deep Diversity: Extensive Variation in the Components of Complex Visual Systems across Animals
Source: Cells. 2022 Dec 8;11(24):3966. doi: 10.3390/cells11243966 (PMC9776813; doi:10.3390/cells11243966)
Supplement: Supplementary file 1 [file cells-11-03966-s001.zip › Supplement/Supplemental_Figures_Deep_Diversity.pdf]

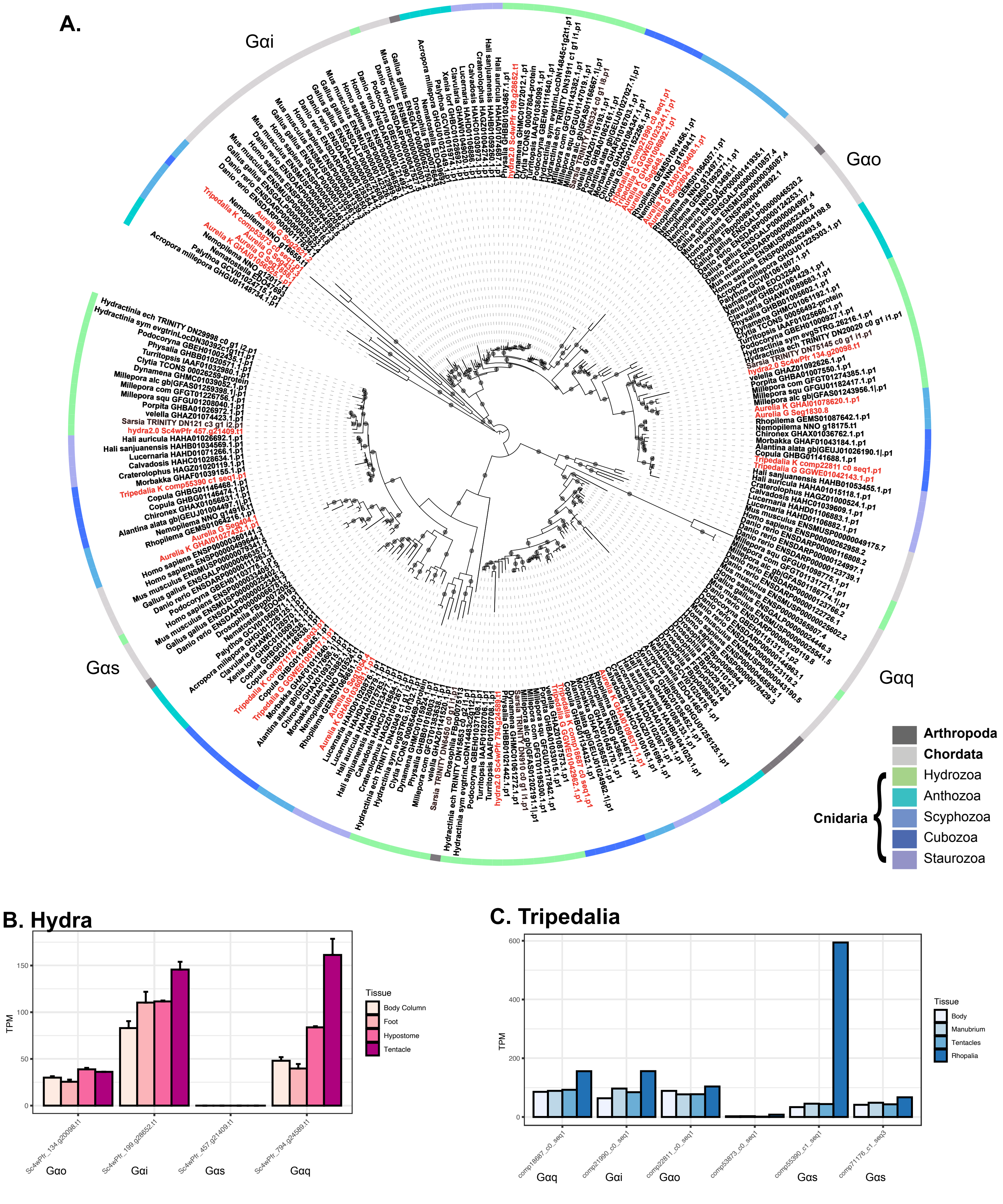

Figure S1. Gα phylogeny and TPM expression plots. A) Gα phylogenetic tree with Hydra, Aurelia, and Tripedalia highlighted in red. B) Expression of Gα genes in Hydra. C) Expression of Gα genes in Tripedalia.

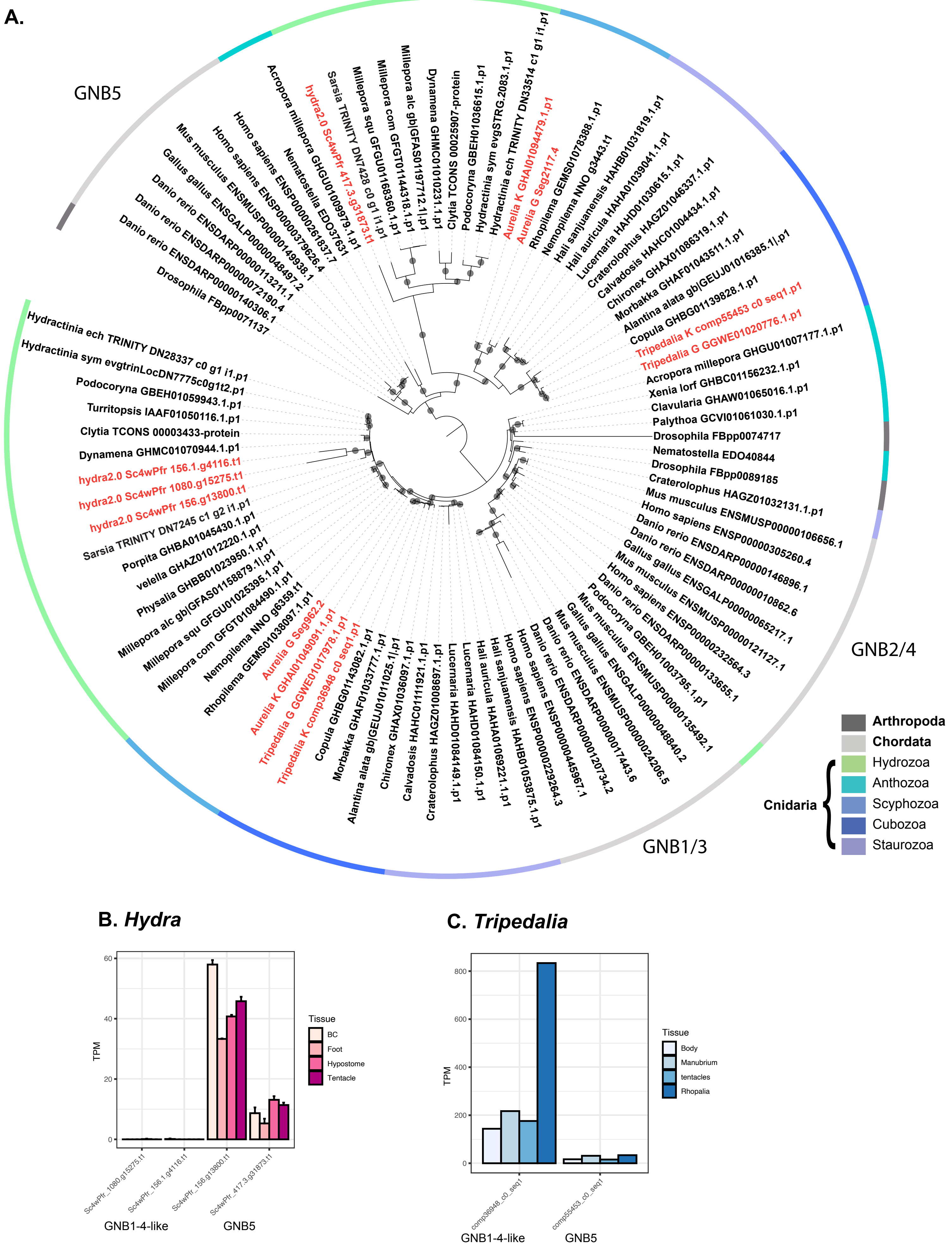

Figure S2. GNB phylogeny and TPM expression plots. A) GNB phylogenetic tree with Hydra, Aurelia, and Tripedalia highlighted in red. B) Expression of GNB genes in Hydra. C) Expression of GNB genes in Tripedalia.

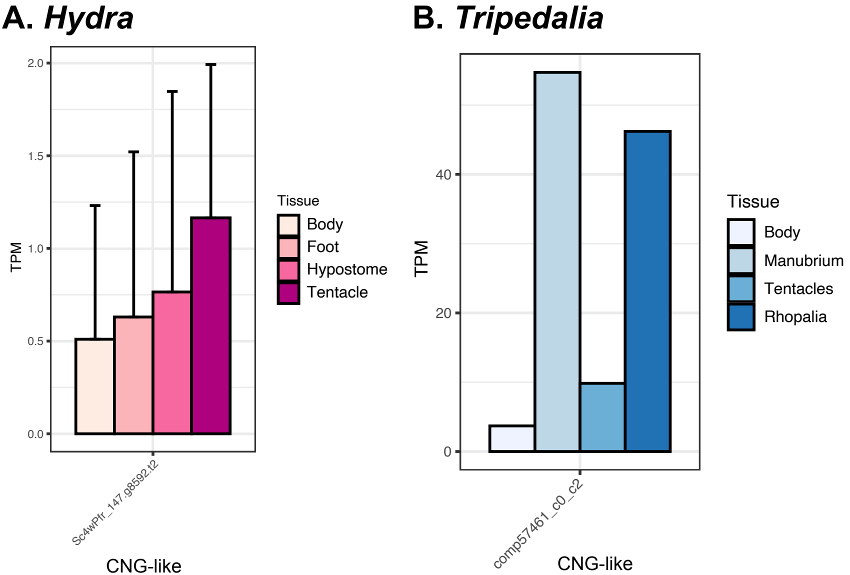

Figure S3. CNG expression plots. A) Expression of CNG-like in Hydra. B) Expression of CNG-like in Tripedalia.

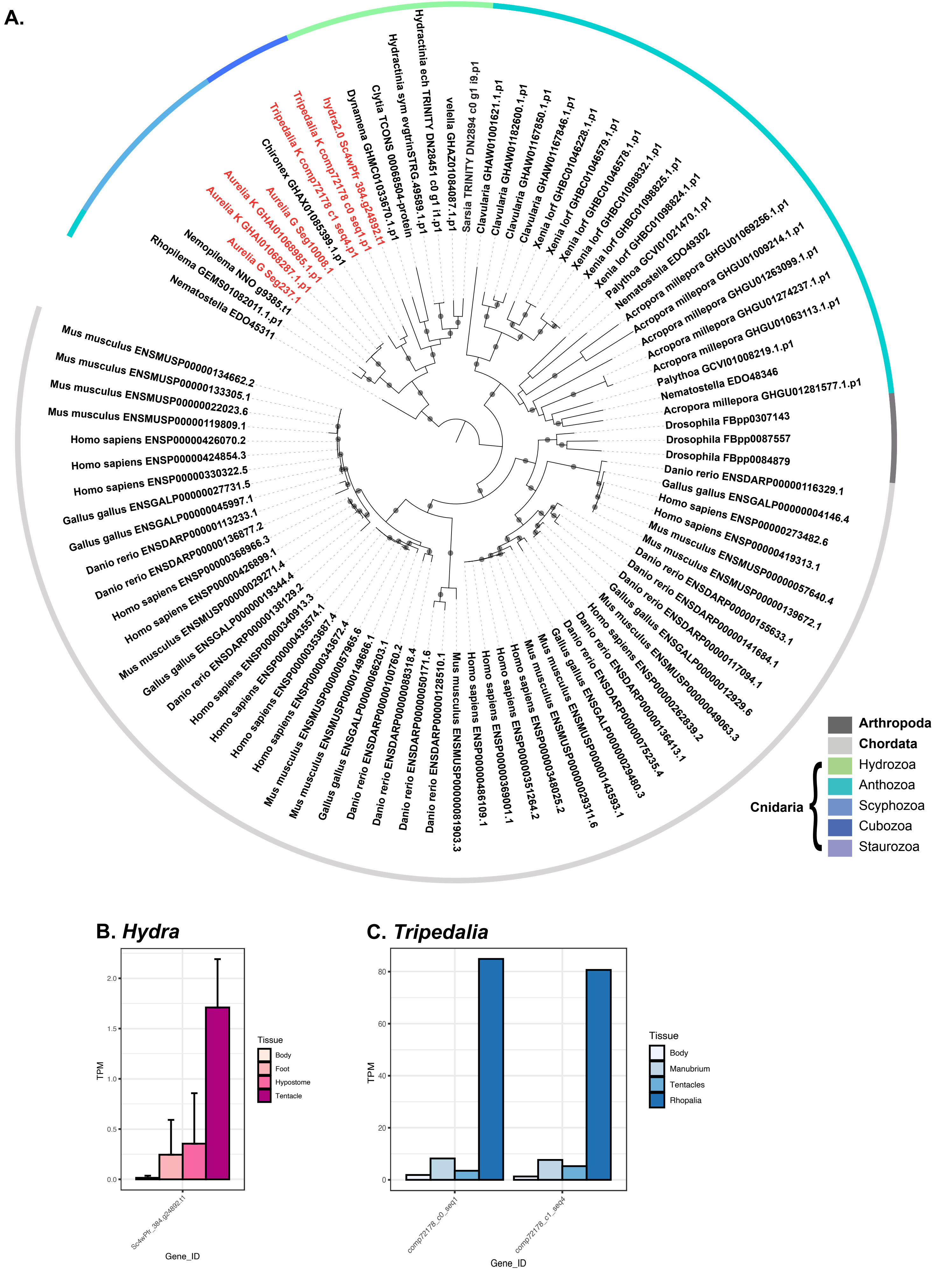

Figure S4. TRP phylogeny and TPM expression plots. A) TRP phylogenetic tree with Hydra, Aurelia, and Tripedalia highlighted in red. B) Expression of TRP in Hydra. C) Expression of TRP in Tripedalia.

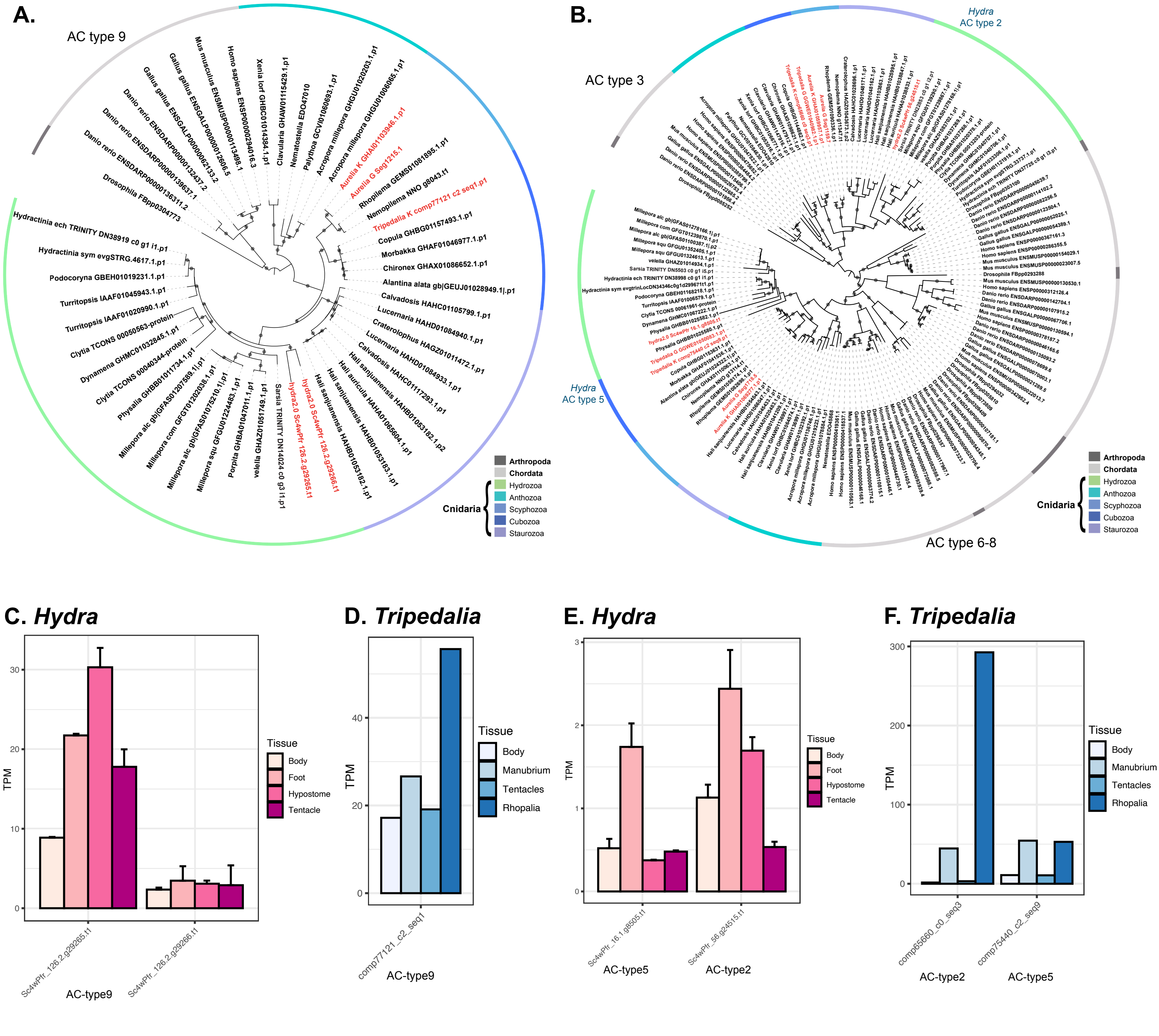

Figure S5. AC phylogenies and TPM expression plots. A) AC-type9 phylogenetic tree with Hydra, Aurelia, and Tripedalia highlighted in red. B) AC-type2-8 phylogenetic tree with Hydra, Aurelia, and Tripedalia highlighted in red. C) Expression of AC-type9 in Hydra. D) Expression of AC-type9 in Tripedalia. E) Expression of AC-type2 and AC-type5 in Hydra. F) Expression AC-type2 and AC-type5 in Tripedalia.

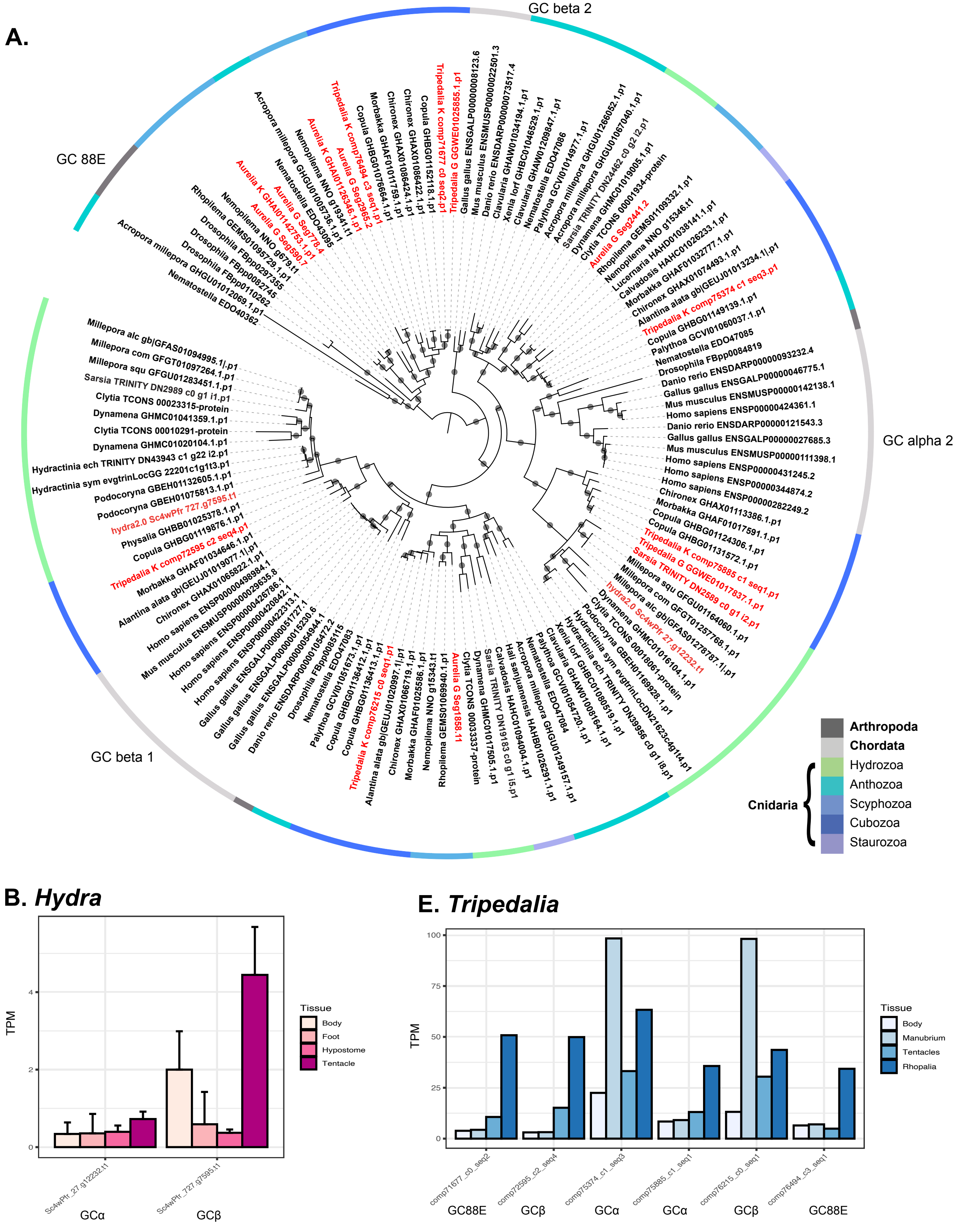

Figure S6. GC phylogeny and TPM expression plots. A) GC phylogenetic tree with Hydra, Aurelia, and Tripedalia highlighted in red. B) Expression of GC genes in Hydra. C) Expression of GC genes in Tripedalia.

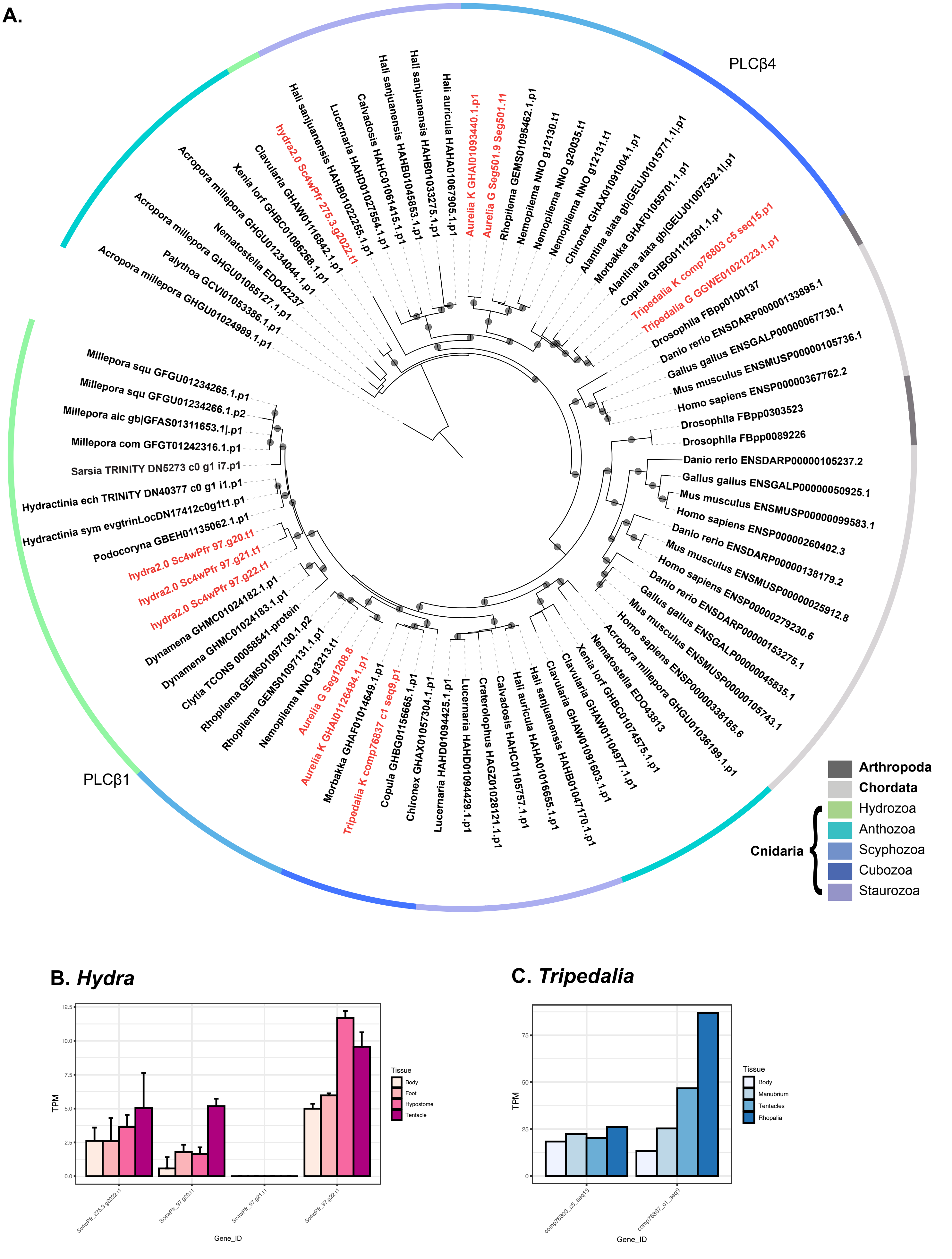

Figure S7. PLC phylogeny and TPM expression plots. A) PLC phylogenetic tree with Hydra, Aurelia, and Tripedalia highlighted in red. B) Expression of PLC genes in Hydra. C) Expression of PLC genes in Tripedalia.

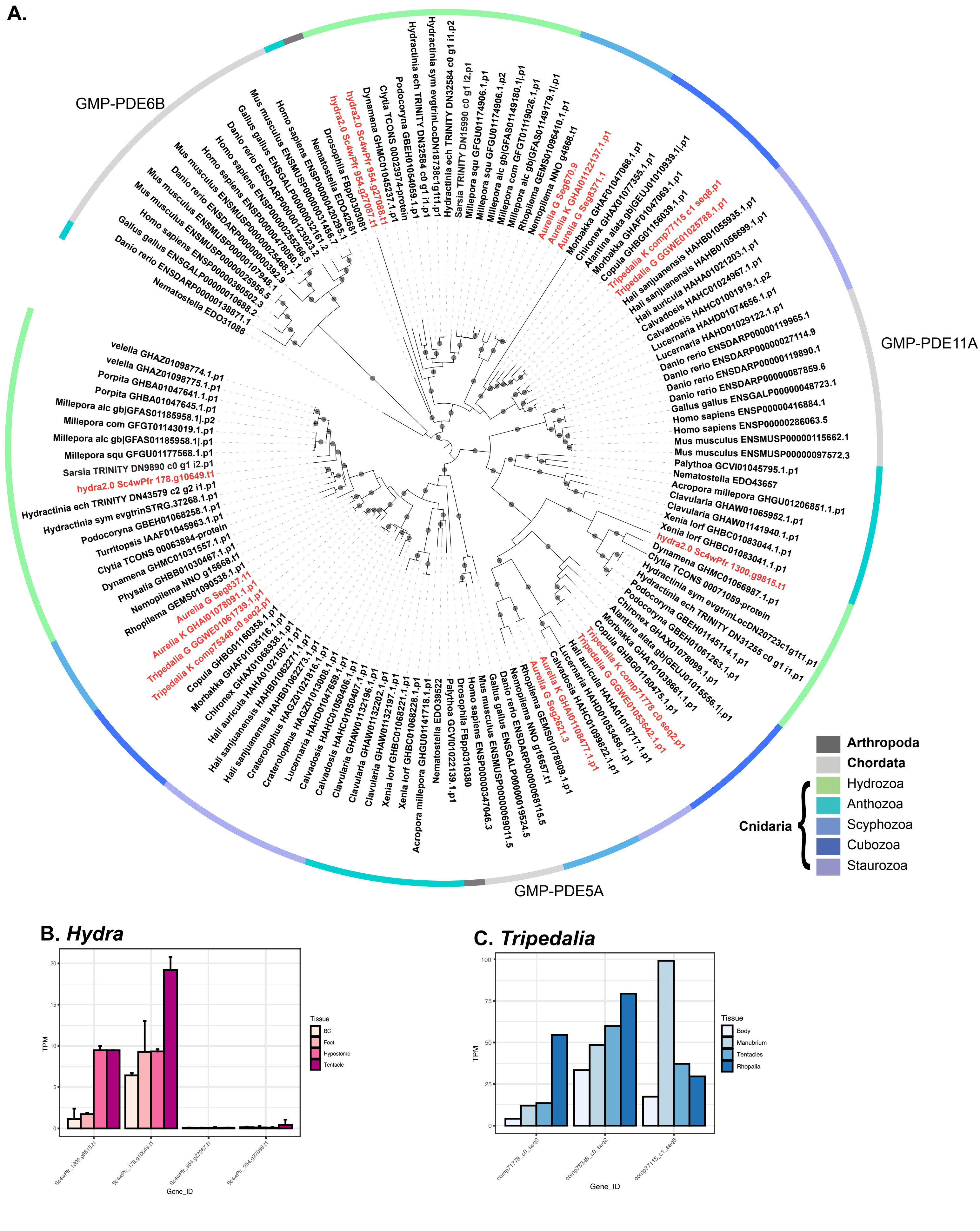

Figure S8. GMP-PDE phylogeny and TPM expression plots. A) PDE phylogenetic tree with Hydra, Aurelia, and Tripedalia highlighted in red. B) Expression of PDE genes in Hydra. C) Expression of PDE genes in Tripedalia.

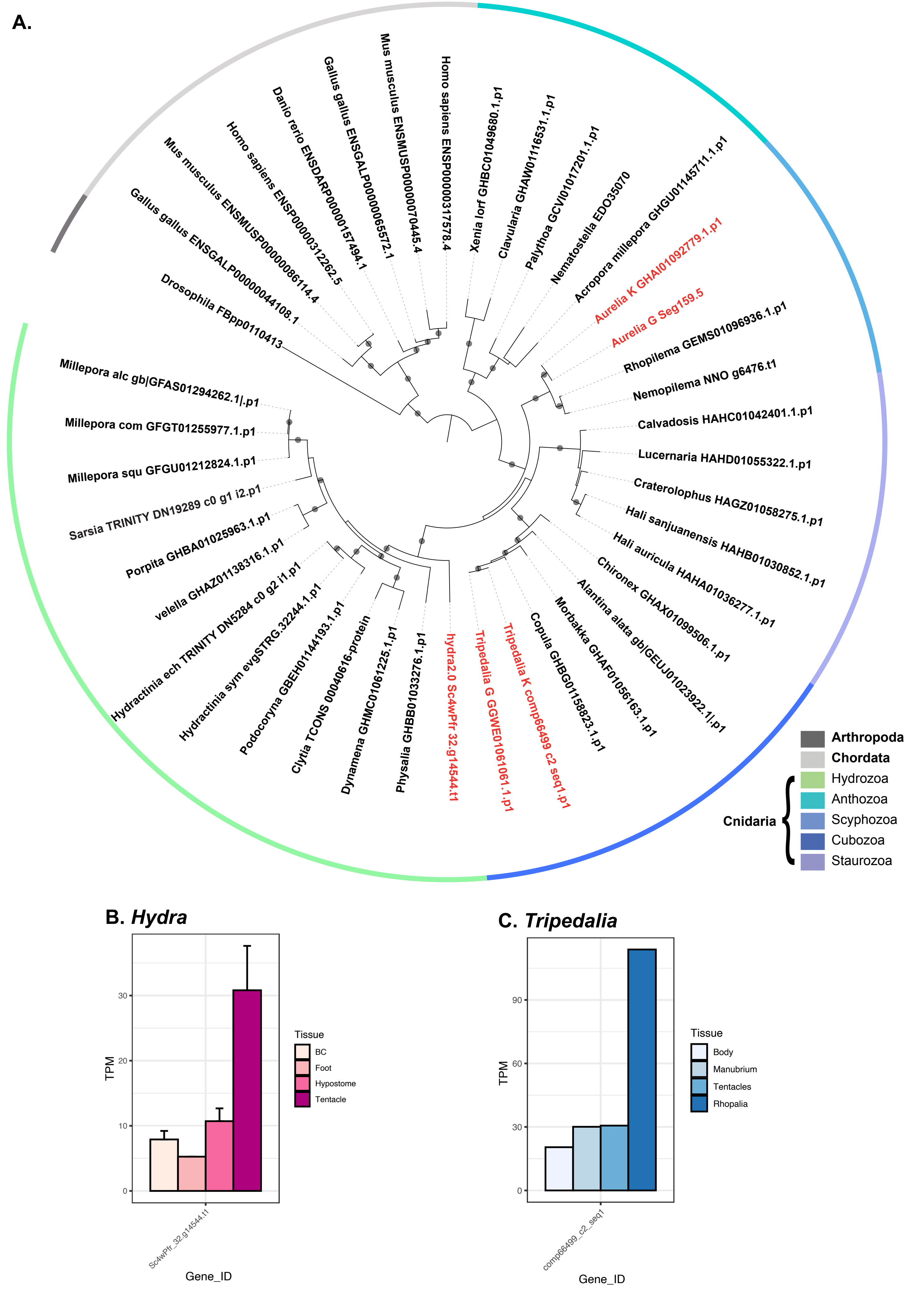

Figure S9. Rhk phylogeny and TPM expression plots. A) Rhk phylogenetic tree with Hydra, Aurelia, and Tripedalia highlighted in red. B) Expression of Rhk in Hydra. C) Expression of Rhk in Tripedalia.

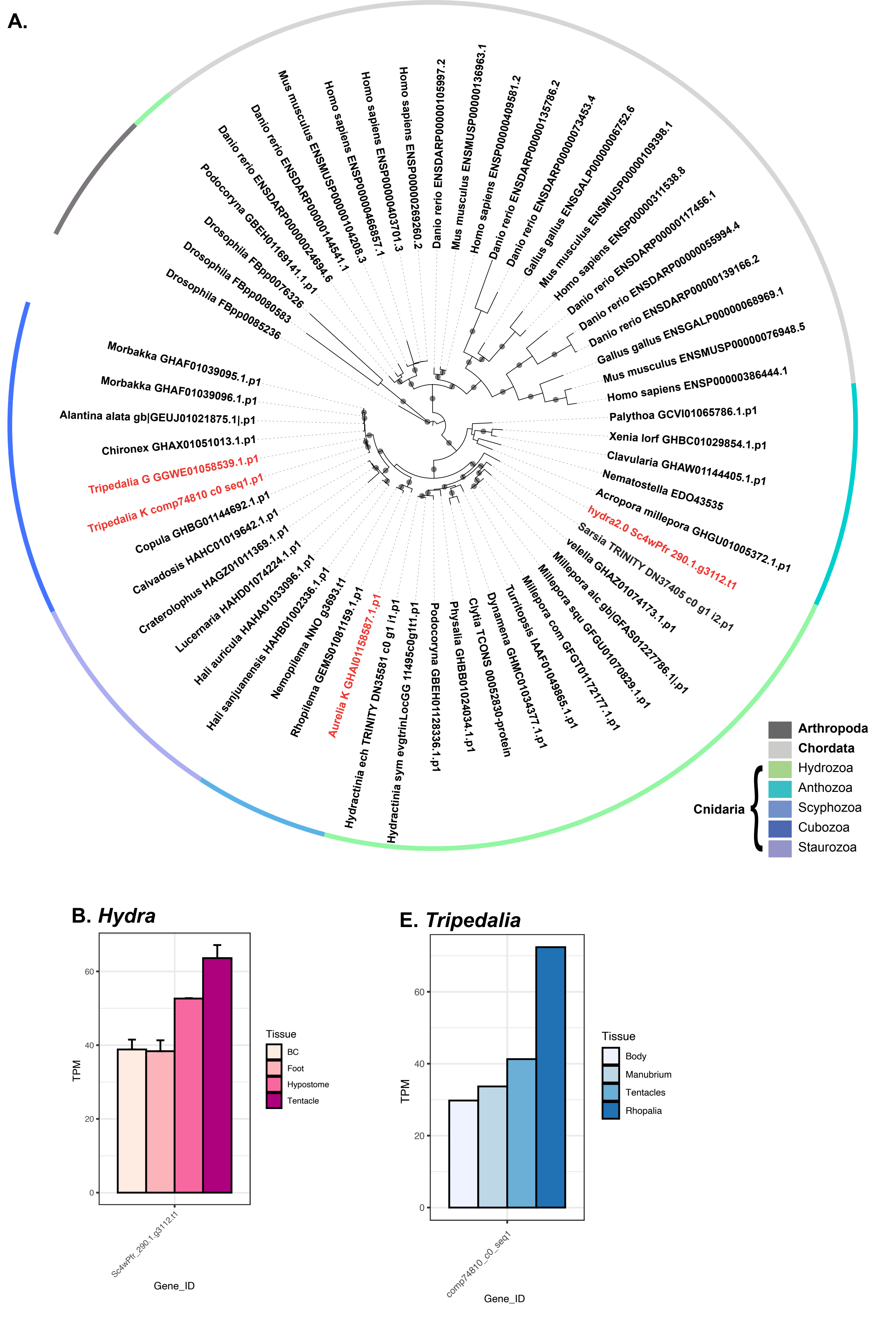

Figure S10. Arrestin phylogeny and TPM expression plots. A) Arrestin phylogenetic tree with Hydra, Aurelia, and Tripedalia highlighted in red. B) Expression of Arrestin in Hydra. C) Expression of Arrestin in Tripedalia.

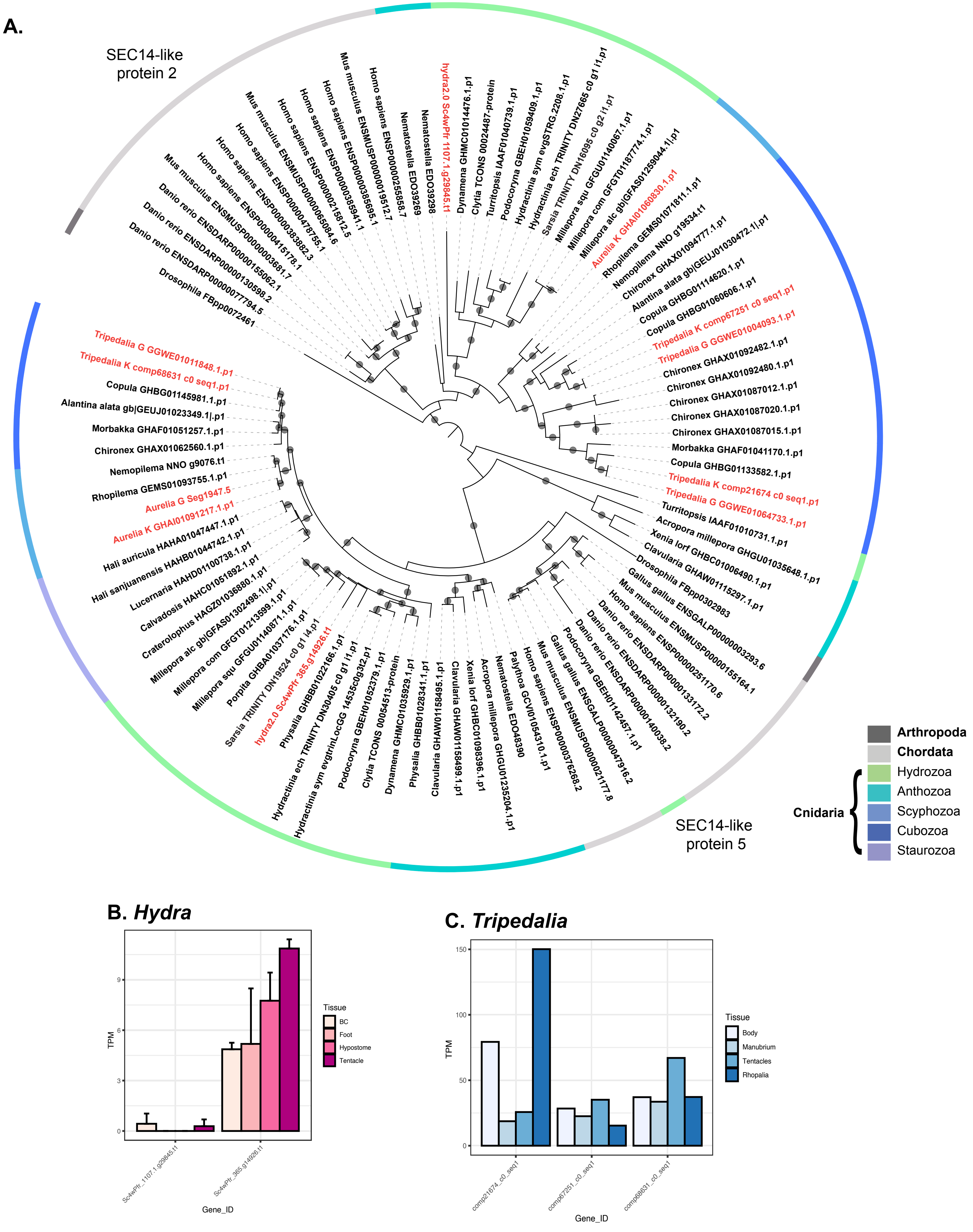

Figure S11. SEC14 phylogeny and TPM expression plots. A) SEC14 phylogenetic tree with Hydra, Aurelia, and Tripedalia highlighted in red. B) Expression of SEC14 genes in Hydra. C) Expression of SEC14 genes in Tripedalia.

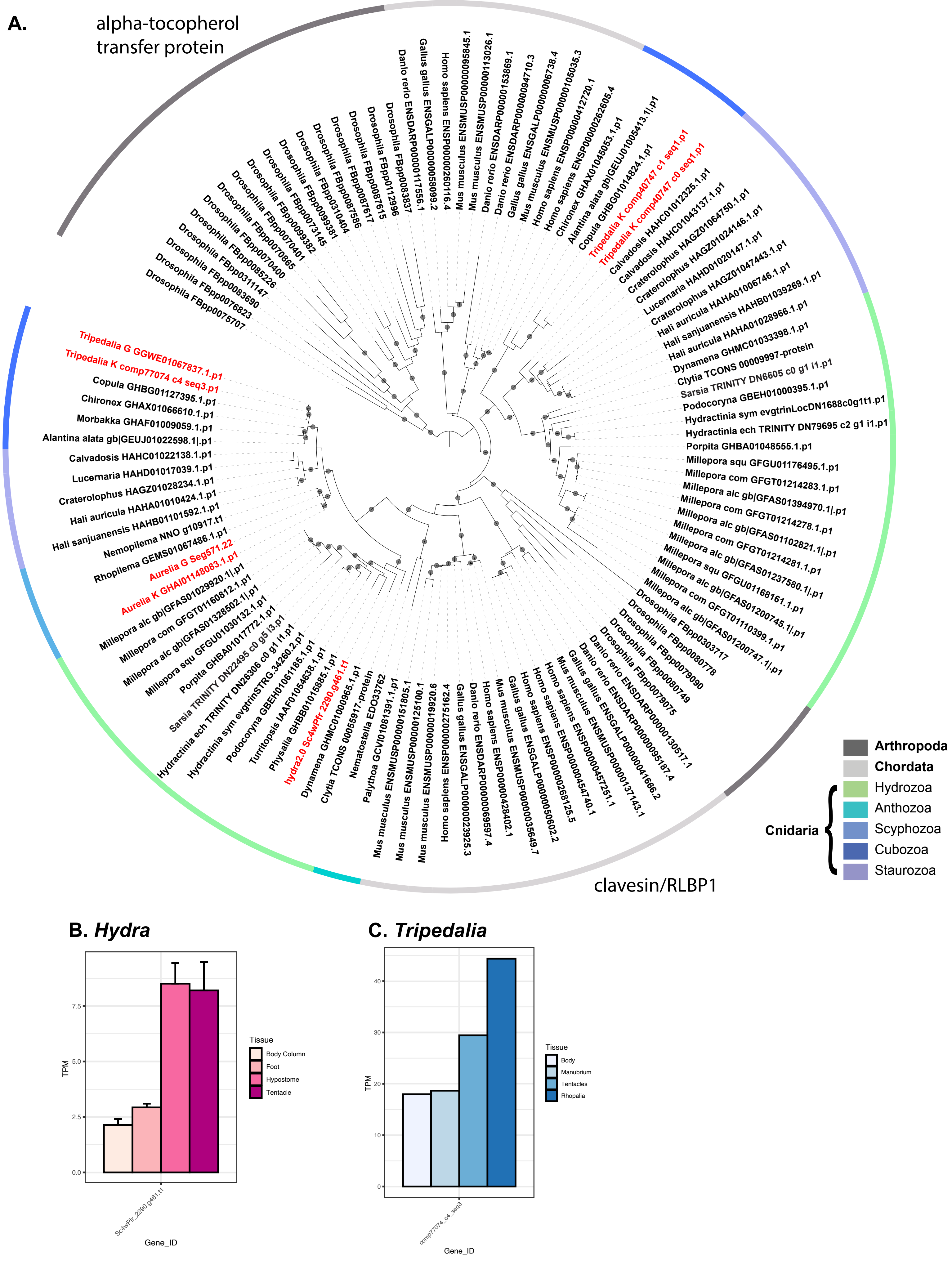

Figure S12. Clavesin/RLBP1 phylogeny and TPM expression plots. A) Clavesin phylogenetic tree with Hydra, Aurelia, and Tripedalia highlighted in red. B) Expression of Clavesin/RLBP1 in Hydra. C) Expression of Clavesin/RLBP1 in Tripedalia.

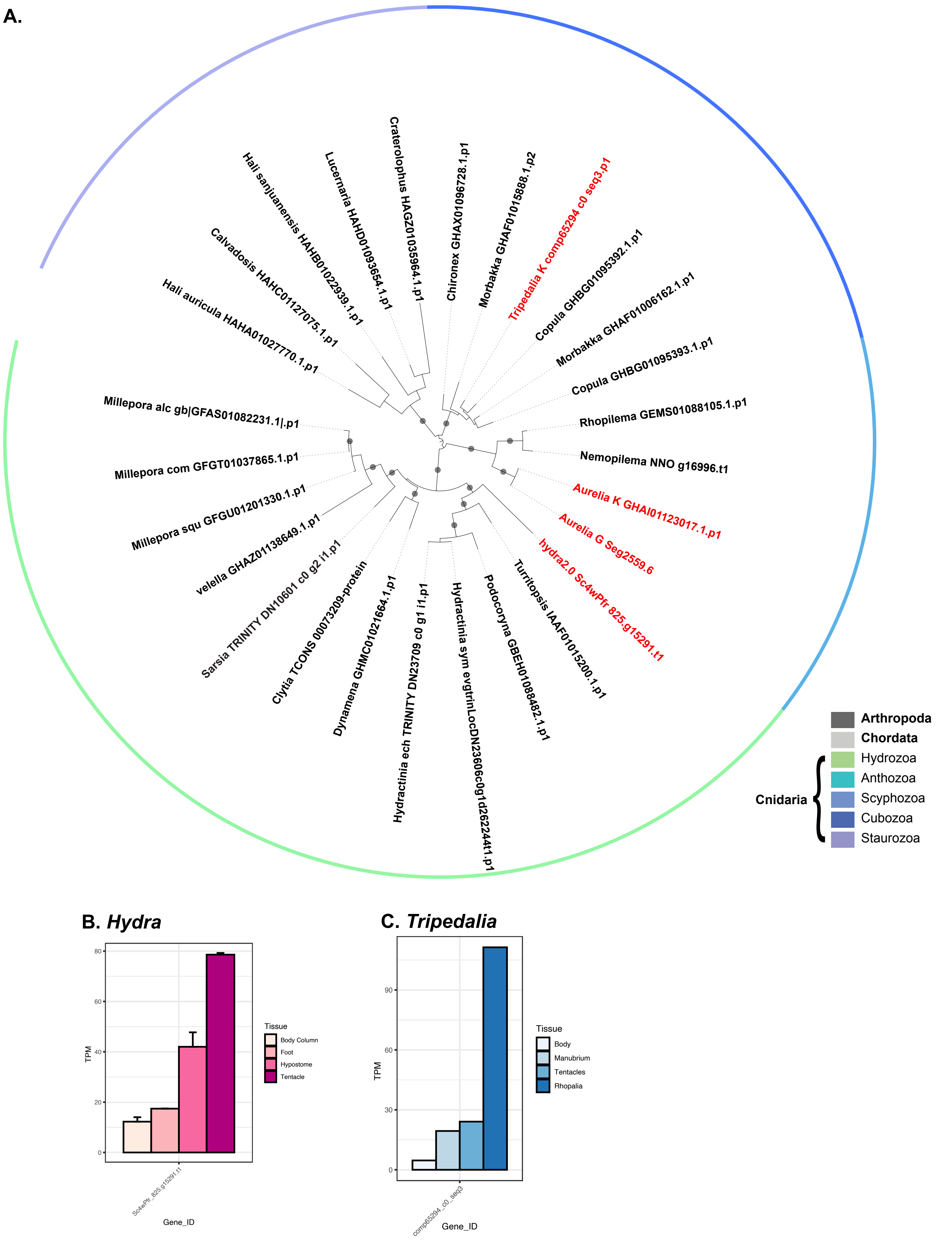

Figure S13. RLBP1-like phylogeny and TPM expression plots. A) RLBP1-like phylogenetic tree with Hydra, Aurelia, and Tripedalia highlighted in red. B) Expression of RLBP1-like in Hydra. C) Expression of RLBP1-like in Tripedalia.

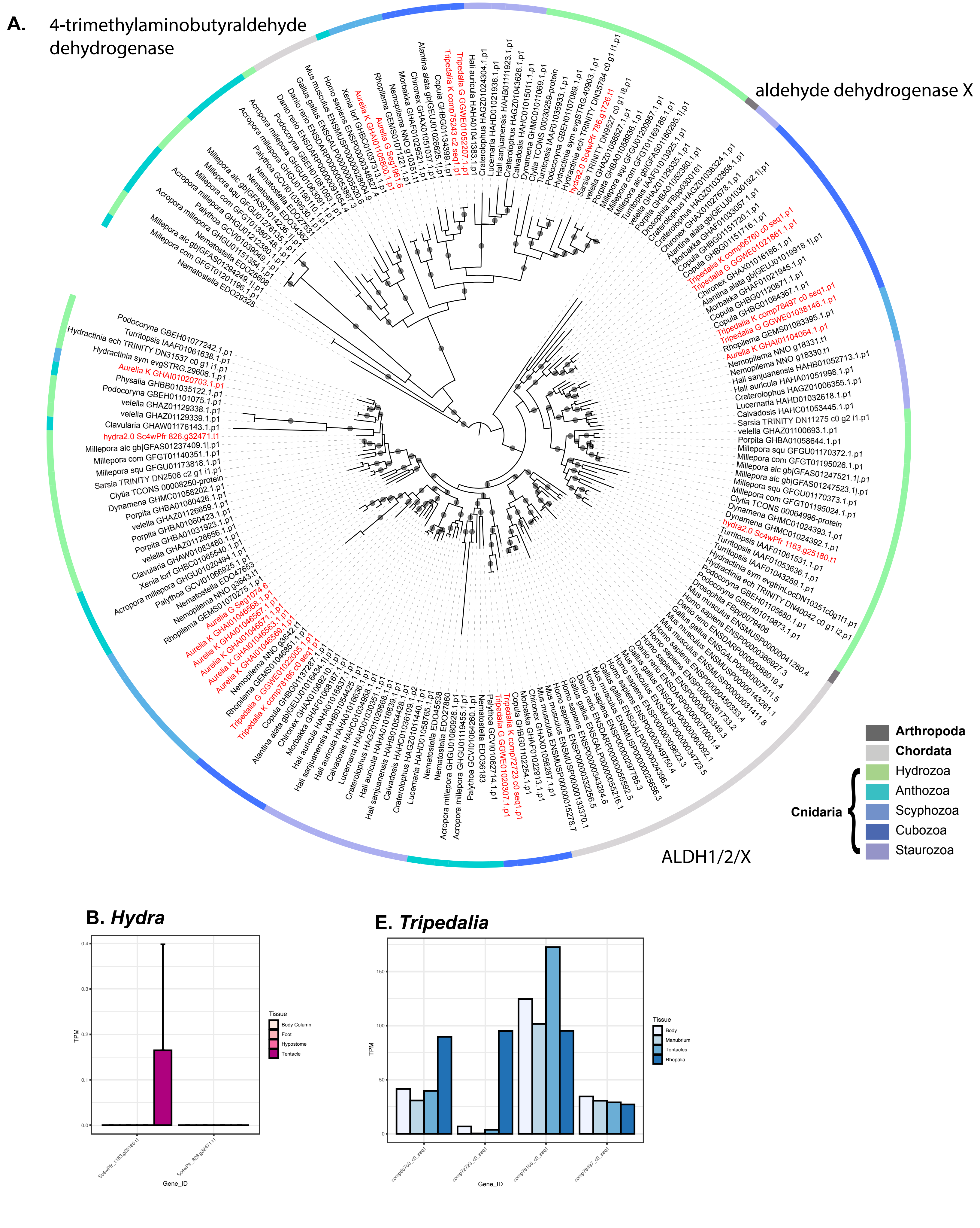

Figure S14. ALDH phylogeny and TPM expression plots. A) ALDH phylogenetic tree with Hydra, Aurelia, and Tripedalia highlighted in red. B) Expression of ALDH genes in Hydra. C) Expression of ALDH genes in Tripedalia.
